# Bing-Neel Syndrome: An Initial Manifestation of Waldenstrom Macroglobulinemia

**DOI:** 10.7759/cureus.19402

**Published:** 2021-11-09

**Authors:** Matthew S Lee, Sanjivani Sathe, Srijan Valasapalli, Maria Grosse Perdekamp

**Affiliations:** 1 Medicine, Carle Illinois College of Medicine, Carle Foundation Hospital, Urbana, USA; 2 Internal Medicine, Carle Foundation Hospital, Urbana, USA; 3 Cancer Center, Carle Foundation Hospital, Urbana, USA

**Keywords:** bing-neel syndrome, waldenstrom macroglobinaemia, myd88l265p, cns lymphoma, lymphoplasmacytic, iwwm-8

## Abstract

Waldenstrom macroglobulinemia (WM) is a low-grade B-cell lymphoma characterized by bone marrow infiltration by monoclonal lymphoplasmacytic cells plus an IgM monoclonal gammopathy. Bing-Neel syndrome (BNS) is a rare manifestation of WM where malignant lymphoplasmacytic cells infiltrate the central nervous system (CNS). Though only present in 0.8% of WM cases, it is likely underdiagnosed and may present before or during WM treatment. Here, we present a case of BNS as an initial sign of WM. A 75-year-old male presented with confusion, gait instability, and expressive aphasia. MRI demonstrated a 5.5-cm mass in the right frontal lobe, crossing midline. Brain biopsy showed CNS lymphoma and later tested positive for the MYD88L265P mutation suggesting WM (as is a mutation in 90-95% WM patients). Indeed, quantitative serum immunoglobulins showed elevated IgM. Initial treatment for WM was started with rituximab, methylprednisolone, carfilzomib, and ibrutinib. MRI two months after initiation showed good response, and the patient was transitioned to ibrutinib monotherapy. Surveillance MRI one year later showed patchy right frontal lobe enhancement indicating disease progression, and therefore the patient was placed back on his initial treatment regimen. However, ibrutinib later had to be held due to thrombocytopenia. Two months after re-starting chemotherapy, he presented with bizarre behavior, and MRI showed extensive disease progression. He was then transitioned to palliative chemotherapy with high-dose methotrexate and rituximab. He has responded well to this regimen, and MRI two years after diagnosis showed no recurrent disease. BNS is a rare but easily missed manifestation of WM. As per the recent National Comprehensive Cancer Network (NCCN) guidelines and the 8th International Workshop on WM (IWWM-8), no standardized diagnostic or management guidelines for BNS is available. Direct brain biopsy is the gold standard for diagnosis. Due to its low incidence, rarity, and limited prospective trial, there is a lack of a clear standard of care therapy. Specific treatment regimen depends on the patient factors and treatment tolerability. IWWM-8 suggests the use of a variety of cytotoxic chemotherapies or ibrutinib. A high-quality meta-analysis of existing reports is critical to characterize the diagnostic features and optimal treatment for BNS. The prognosis of BNS remains unclear, with an estimated three- and five-year survival rate at 59% and 71%, respectively. BNS is an infrequent complication of WM. Clinicians should suspect BNS with persistent, unexplained neurologic symptoms in WM.

## Introduction

Waldenstrom macroglobulinemia (WM) is an uncommon but well-known syndrome involving an IgM monoclonal gammopathy in the context of a lymphoplasmacytic lymphoma (LPL). In the majority of WM cases, the LPL invades the bone marrow. However, in rare instances, the malignant plasmacytoid lymphocytes can invade the central nervous system (CNS). This constitutes the Bing-Neel syndrome (BNS). Interestingly, BNS was first reported eight years before WM was described [1-2].

While estimated to be present in only 0.8% of WM cases [3], BNS presents with nonspecific symptoms that may be hard to distinguish from conventional WM. It may present in patients with no history of WM or represent disease progression in a previously stable WM or persistent unexplained neurological symptoms in a patient with WM. In this report, we describe a rare case of BNS as the initial manifestation of WM.

## Case presentation

**Figure 1:**
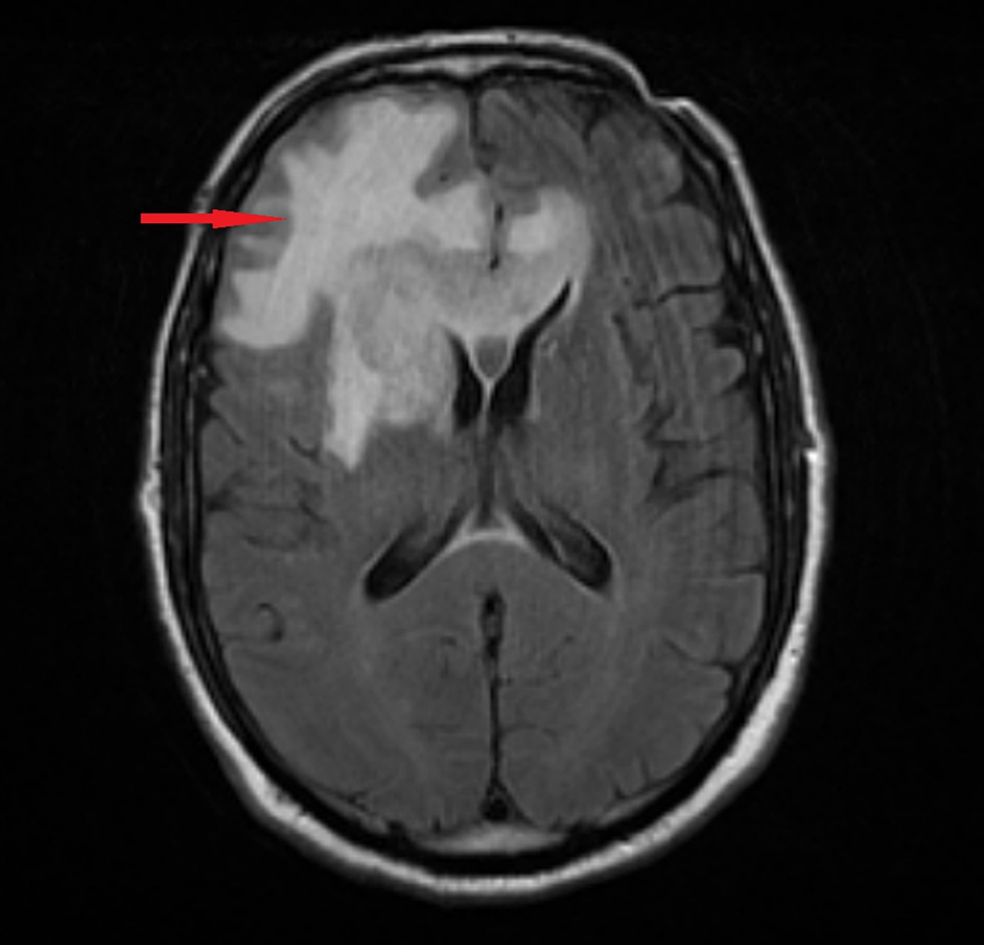
Enhancing intra-axial mass measuring 5.5 cm x 3.6 cm x 3.2 cm is predominantly located in the deep white matter of the right frontal lobe and also crosses midline (via the genu of the corpus callosum). Mass demonstrates DWI hyperintense signal, consistent with hypercellularity. DWI, diffusion-weighted imaging

A 75-year-old male presented to our hospital with worsening mental status. Gait instability and expressive aphasia were noted on the physical examination. He was otherwise hemodynamically stable, with unremarkable laboratory studies and a negative urine drug screen. Computed tomography (CT) of the head without contrast showed a large right frontoparietal lesion crossing midline with surrounding vasogenic edema (Figure 1). Further characterization with magnetic resonance imaging (MRI) showed a 5.5-cm intra-axial mass within the deep white matter of the right frontal lobe, with gadolinium enhancement and prominent FLAIR (fluid-attenuated inversion recovery) signal concerning malignancy.

**Figure 2:**
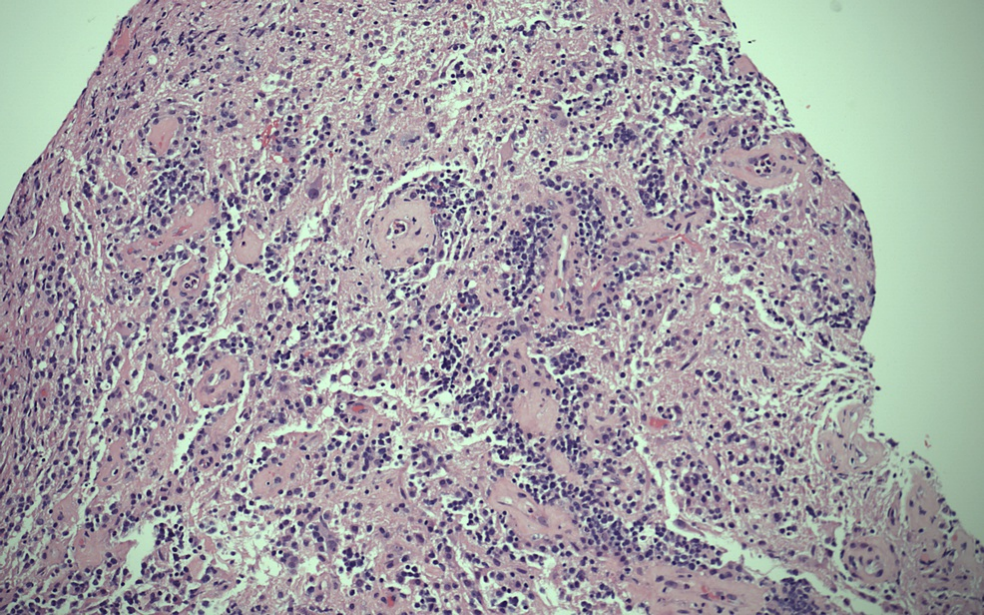
H&E 10x: cells have vesicular chromatin and membrane-bound nucleoli. H&E, hematoxylin and eosin

**Figure 3:**
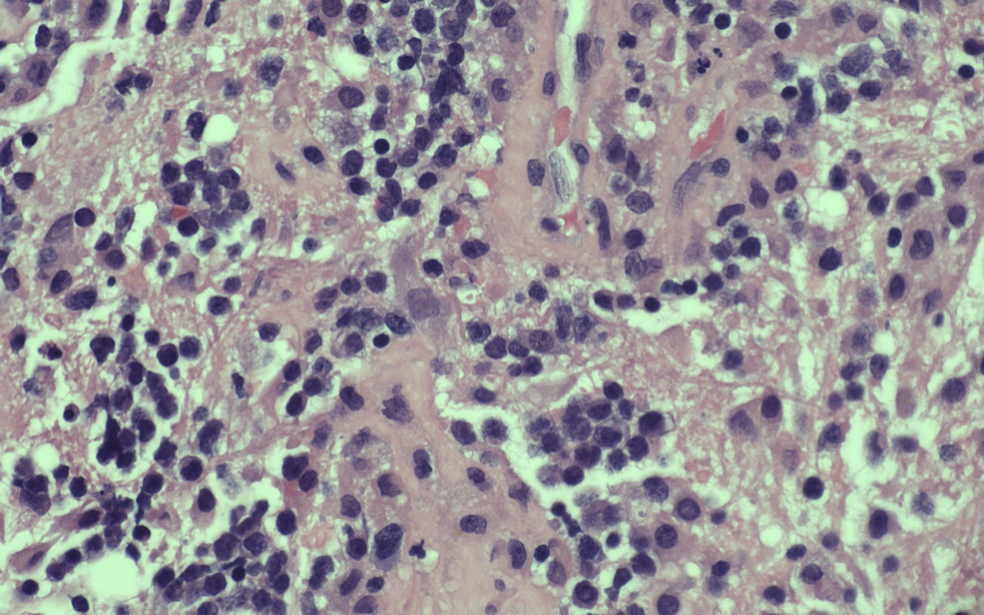
H&E 20x: cells are round, hyperchromatic and with prominent nucleoli. H&E, hematoxylin and eosin

**Figure 4:**
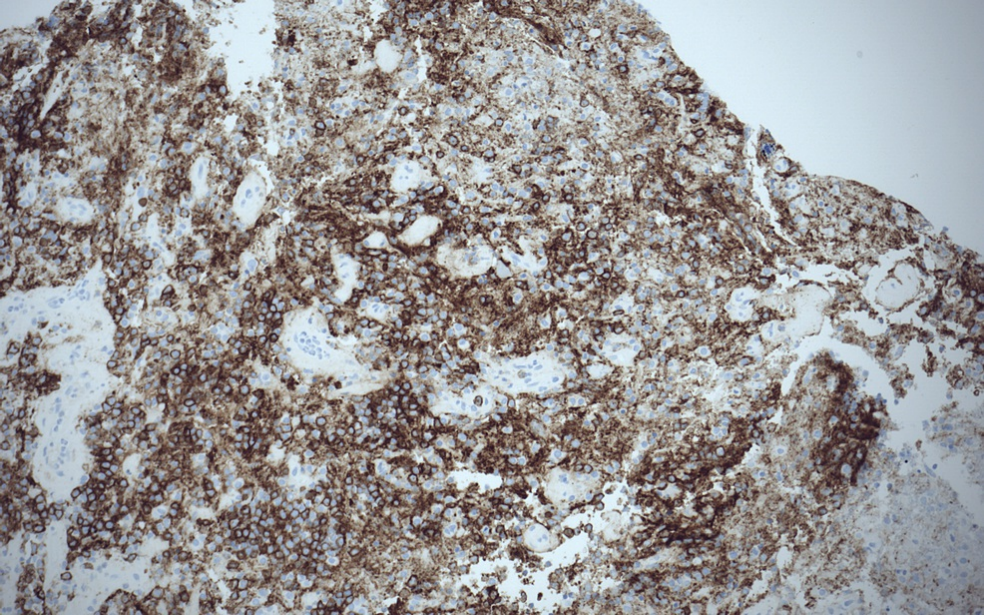
Cells positive for CD20 immunostain.

Later, the patient was started on dexamethasone, and a frontal stereotactic brain biopsy was performed. The initial pathology report showed a B-cell lymphoma with cells positive for CD20 immunostain (Figures 2-4). However, due to its complexity, the sample was sent to an outside institution for a second opinion, which found the sample to be positive for the MYD88L265P mutation. Due to the strong association between this mutation and WM, quantitative serum immunoglobulins were measured, which showed an elevated IgM monoclonal paraprotein, confirming the diagnosis of WM.

**Figure 5:**
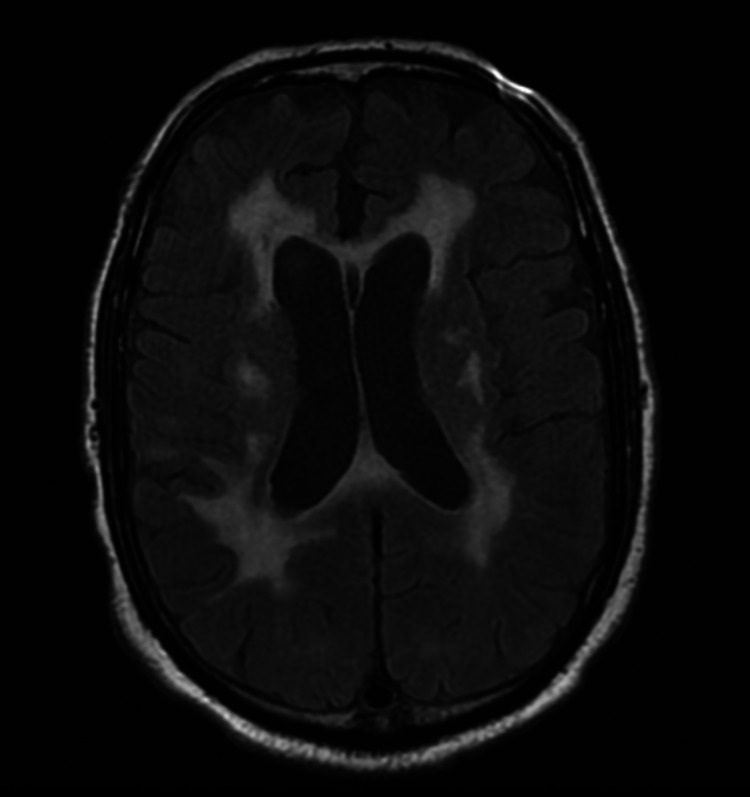
Repeat MRI in a year showing extensive abnormal FLAIR signal involving the bilateral cerebral hemispheres and no evidence of an abnormal enhancing intracranial mass. FLAIR, fluid-attenuated inversion recovery

The patient was started on treatment with rituximab, methylprednisolone, carfilzomib, and ibrutinib. The patient showed a good response by MRI at three months, and he was transitioned to oral ibrutinib only. Surveillance MRI at one year post-diagnosis showed patchy right frontal lobe enhancement indicating disease progression, although the patient had no clinical symptoms; he was then restarted on the initial chemo-immunotherapy protocol. However, ibrutinib was later held due to thrombocytopenia. Soon after, the patient presented to the hospital again with cognitive dysfunction, leg weakness, and falls, with MRI showing significant disease progression. At this point, his treatment was changed to palliative chemotherapy with high-dose methotrexate and rituximab. His disease responded avidly to this regimen, and he is currently completing a one-year course of treatment, with no radiologic or clinical evidence of recurrence (Figure 5).

## Discussion

BNS is a rare form of WM in which malignant lymphoplasmacytic cells invade the CNS. The clinical presentation encompasses a broad range of neurologic symptoms. The most commonly reported are balance and gait abnormalities, cranial nerve palsies, limb motor deficits, and altered mental status [4-6]. Other presentations include peripheral neuropathy and other sensory deficits, headache, and, in the case of tumoral lesions, seizures, and focal neurologic deficits. The CNS involvement is usually diffuse (74%-93% of cases) [6], with infiltration of the leptomeninges, periventricular white matter, and/or spinal cord. In a minority of cases, such as ours, BNS may present as a single mass [7].

There are currently no standardized diagnostic or management guidelines for BNS. Most data come from single patient cases or retrospective case series. In 2017, the 8th International Workshop on WM proposed practical guidelines for diagnosing and treating BNS. For diagnosis, the consortium recommended histologic evidence of LPL on CNS biopsy, or molecular and cytologic evidence on CSF analysis [5]. Imaging is an important corroborative study but is not diagnostic in isolation. Roughly 80% of BNS cases show MRI abnormalities, most commonly leptomeningeal enhancement in the brain and spinal cord [4-6]. However, there is no pathognomonic finding, and a normal MRI does not exclude the diagnosis [5].

Discovering the MYD88L265P in our patient’s biopsy sample was the first clue that his disease was not a routine CNS lymphoma [8]. Indeed, the MYD88L265P has been found in >90% of cases of WM8. However, false-positives can occur, such as if the CNS sample were contaminated with peripheral blood. The MYD88L265P mutation is also present in a significant minority of diffuse large B-cell lymphomas [9]. Thus, the presence of the MYD88L265P mutation in CSF is sensitive but not specific for BNS. Nonetheless, the discovery of this mutation in a patient does warrant a careful evaluation for WM.

There is not yet sufficient evidence to recommend one specific first-line treatment for BNS. High-dose methotrexate or cytarabine, purine analogues, and bendamustine have all shown efficacy [5]. Rituximab is a popular adjunctive immunotherapy, although there is conflicting evidence about both its efficacy [4-6] and CNS penetration [10]. The Bruton tyrosine kinase (BTK) inhibitor ibrutinib has become a popular choice among practitioners. In a study of 28 BNS patients treated with ibrutinib, 85% showed symptomatic improvement [11]. Interestingly, although one case report describes a patient with BNS refractory to methotrexate and rituximab who responded avidly to ibrutinib [12], our patient followed the opposite sequence. His initial response to ibrutinib eventually gave way to disease relapse, which has since been under control with methotrexate and rituximab. This suggests that it is reasonable to switch to another regimen in the case of nonresponse or relapse. Second-generation BTK inhibitors have demonstrated safety and efficacy for WM patients [13-14] and may prove useful in treating BNS as well. Finally, autologous stem cell transplant has achieved remarkable results in a small case series, where 11/14 patients with BNS showed disease response and 10/14 remained in remission at three years [15]. The prognosis of BNS remains unclear; a 34-patient series estimated three-year survival at 59% [4], whereas a 44-patient series estimated five-year survival at 71% [6].

## Conclusions

Although rare, BNS is likely underdiagnosed. Its nonspecific neurologic symptoms may mimic the hyperviscosity syndrome and peripheral neuropathy seen in conventional WM. However, new, persistent, and asymmetric symptoms are atypical of WM alone and should raise suspicion for BNS. Clinicians must remain vigilant, as BNS may be the only sign of WM progression in a patient well-controlled on treatment. Furthermore, as in our patient, up to one-third of cases of BNS cases can present as the initial manifestation of WM.

Radiologic features and the MYD88L265P mutation are important clues pointing toward BNS, but definitive diagnosis requires CNS biopsy or cerebrospinal fluid (CSF) analysis. There remains no gold-standard treatment regimen for BNS. Due to its rarity, a randomized controlled trial is likely not feasible. Therefore, a high-quality meta-analysis of existing reports will be valuable to determine the optimal treatment.
